# Microbiological quality and proximate analysis of locally produced soymilk drinks sold in Calabar Metropolis; a public health assessment

**DOI:** 10.4314/ahs.v23i3.87

**Published:** 2023-09

**Authors:** G E John, E A Okpo, J Akpanke, C U Okoro, P A Omang, J A Lennox

**Affiliations:** Department of Microbiology, Faculty of Biological Sciences, University of Calabar, Nigeria

**Keywords:** Soymilk, contamination, public health, hygiene, foodborne illnesses

## Abstract

**Background:**

This study was aimed at evaluating the microbial quality and proximate analysis of soymilk produced and sold within Calabar Metropolis.

**Methodology:**

Soymilk samples were purchased in pairs from five locations in Calabar Metropolis. The samples collected were subjected to microbiological and proximate analysis to ascertain the hygienic standards of the products and nutrient contents.

**Results:**

The result showed that the highest heterotrophic bacterial count of 5.3 × 10^5^ cfu/ml was recorded in soymilk sample sold in Goldie market while the least (2.7 × 10^4^ cfu/ml) was recorded at Watt market. The mean fungal count was highest in Akim market samples (5.8 x 10^4^ cfu/ml) and lowest in Atimbo market samples (2.4 x 10^3^ cfu/ml). The probable bacterial and fungal isolates were identified as: *Pseudomonas aeruginosa, Bacillus* sp, *Escherichia coli*, Klebsiella sp, *Salmonella* sp, *Streptococcus* sp, *Staphylococcus aureus, Aspergillus* sp, *Rhizopus* sp, *Penicillium* sp and *Mucor* sp. All the isolated bacteria species were found to be resistant to more than 50% of the antibiotics used. Proximate composition analysis of the soymilk samples revealed high moisture, carbohydrate and protein contents.

**Conclusion:**

The microbial population detected in terms of number and types reflected poor hygienic standard of production.

## Introduction

Soymilk is a healthy satisfying beverage obtained from the water extract of soybeans. It has a smooth creamy texture with an off-white emulsion-like suspension containing proteins, water soluble vitamins and carbohydrates^1^. Soyabean is a substrate used for the production of a number of fermented products such as fermented beverages, culture drinks, yoghurt-like products, frozen desserts, cheese substitutes, tofu, sauces and other typical Asian products. Microorganisms found in soymilk contribute to its spoilage. In addition to poor handling and unhygienic practices of local producers of soymilk products, the nutrient composition of soymilk makes it an excellent bacteriological medium. These have been implicated in the occurrence and prevalence rate of diseases such as typhoid fever and dysentery among soymilk consumers^2^

The consumption of locally produced cereal foods and drinks are becoming very popular, with acceptability cutting across the various multi-ethnic groups and socioeconomic classes. They have economic potentials especially now that emphasis is on development of local foods. The high cost and scarcity of milk supplies in developing countries has led to the development of alternative milk supplies from vegetable sources such as soymilk. This is a vital substitute when solving malnutrition problems in developing countries like Nigeria^1^. These beverages are however prone to microbial spoilage if not properly processed, handled and stored.

Health benefits of soymilk include low lactose and cholesterol content, reduced bone loss, prevention and reduction of heart diseases^3^. Despite this array of benefits derivable from soymilk, previous studies have reported that it can easily be a route for transmitting foodborne illnesses. Foodborne or waterborne microbial pathogens cause diarrheal diseases, which is a leading cause of illness and death in less developed countries, killing an estimated 1.9 million people annually at the global level^4^. This study was therefore aimed at evaluating the microbial quality and nutritive value of soymilk locally produced and sold within Calabar Metropolis.

## Materials and Methods

### Collection of samples

A total of Ten (10) soymilk samples were purchased two each from five locations in Calabar Metropolis namely; Watt market, Akim market, Goldie market, Atimbo market and Marian market. The samples were properly labelled, placed in plastic containers and transported to the Department of Microbiology Laboratory, University of Calabar for analysis.

### Microbiological analysis

The pour plate method of Downes and Ito^5^ was used. One milliliter (1 ml) of desired dilution (10^-4^) was pour plated in duplicates onto plate count agar, eosin methylene blue (EMB) agar and sabouroud dextrose agar (SDA). All the bacteria plates were then incubated at 37°C for 24 hours while the fungi plates were incubated at 25°C for 48-72 hours. After 24 and 72 hours, the bacteria and fungi colonies that appeared on plates were enumerated accordingly. Cultural characteristics of discrete colonies were also observed and noted.

### Characterization and Identification of the isolates

Purified isolates were characterized by gram morphology and biochemical test using Bergey's manual of determinative bacteriology^6^. Fungi isolates were characterized and identified according to their cultural morphology and microscopy using the scheme of Samson and Varga^7^ and Wantanabe^8^. The identification of the fungi was based on the examination of the conidial heads, philiades and conidiosphore.

### Antibiotic sensitivity test of the isolated bacteria

The antibiotic sensitivity testing of the isolated organisms was carried out using disc diffusion assay as described by Baur *et al*^9^ and was interpreted according to the guidelines of Clinical Laboratory Standard Institute^10^.

### Proximate composition of soymilk

Proximate analysis of the soymilk samples was carried out for ash, moisture, crude fibre lipid, protein and carbohydrate content according to the method described by AOAC^11^.

## Results

The microbial load of soymilk sold in Calabar metropolis is presented in Table 1. The highest heterotrophic bacterial count of 5.3 × 105 cfu/ml was observed in soymilk sample sold in the Goldie area while the least (2.7 × 104 cfu/ml) was recorded at Watt area. The mean fungal count was highest in Akim market 5.8 × 104 cfu/ml and lowest in Atimbo area 2.4 × 103 cfu/ml. Biochemical characterization of bacterial isolates from soymilk samples as presented in Table 2 showed that the samples were contaminated with coliforms and other bacterial species. The result revealed a total of 23 distinct bacterial species belonging to 6 different genera. Biochemically, the bacterial isolates were identified to be; *Pseudomonas aeruginosa, Bacillus* sp, *Escherichia coli, Klebsiella* sp, *Salmonella typhi, Streptococcus* sp and *Staphylococcus aureus*. Table 3 showed the cultural morphology and microscopic identification of the fungi isolated from the soymilk samples analyzed in this study. The fungi isolated were *Aspergillus* sp, *Rhizopus* sp, *Penicillium* sp and *Mucor* sp.

**Table 1:** Microbial load of soymilk samples locally produced in Calabar Metropolis

Location	Mean microbial count in CFU/mL		
	THBC	TCC	TFC
Watt market	2.7 x 10^4^	2.9 x 10^2^	6.4 x 10^3^
Akim market	1.1 x 10^5^	4.1 x 10^2^	7.4 x 10^3^
Marian market	9.2 x 10^4^	2.7 x 10^2^	5.8 x 10^4^
Atimbo market	3.8 x 10^4^	3.6 x 10^2^	2.4 x 10^3^
Goldie market	5.3 x 10^5^	5.4 x 10^2^	3.7 x 10^3^

THBC: Total Heterotrophic Bacterial Count; TCC: Total Coliform Count; TFC: Total Fungal Count

**Table 2:** Cultural and morphological characterization of fungal isolates

Number of isolates	Colony morphology	Microscopic morphology	Probable organism
4	Growth begins as yellow colonies that soon develop a black, dotted surface as conidia are produce within 26 days. The colony becomes jet black and powdery and the reverse remains cream color	Exhibits septate hyphae long conidiophores that support spherical vesicle that give rise to mutulae and phalides from which conidia are produce.	*Aspergillus niger*
2	Black fluffy coloration with powdery appearance	Non-septate hyphae with sporangiophores	*Rhizopus* spp
1	Green colonies, surface of colonies becomes powdery due to presence of conidia	Hyphae are septate and produce brush like conidiophores, conidiophores produce metulae from which phalides producing chains of conidia arise	*Penicillium* sp
3	Growth begins as white to grey colonies that later turn to brown	Non septate with a visualized sporangiophores and sporangia. Presence of branched coenocytic hypha and black spores	*Mucor* sp

**Table 3:** Biochemical characterization and identification of the isolates

Number of isolates	Gram's reaction	Cell morphology and arrangement	Catalase	Hemolysis	Motility	Oxidase	Coagulase	Indole	Citrate	H_2_S	MR	VP	Nitrate	Urease	Xylose	Fructose	Raffinose	Glucose	Lactose	Mannose	Sucrose	Mannitol	Maltose	Ribose	Salicin	Probable organism
5	+	Cocci in clusters	+	B	-	-	+	-	+	-	+	+	+	+	-	+	+	+	+	+	+	+	+	+	-	*Staphylococcus aureus*
2	+	Cocci in clusters	-	B	-	-	nt	-	+	-	+	-	+	-	-	+	-	+	+	+	+	-	+	-	+	*Streptococcus* sp
2	-	Rods in singles	+	-	+	+	nt	-	+	-	-	-	+	-	-	-	-	-	-	-	-	+	-	-	+	*Pseudomonas aeruginosa*
1	+	Long rods in pairs	+	nt	+	±	nt	-	+	-	-	+	+	-	+	+	+	+	±	+	+	+	+	+	+	*Bacillus* sp
4	-	Rods in pairs	+	nt	+	-	nt	-	-	+	+	-	-	-	+	+	-	+	-	+	-	+	+	-	-	*Salmonella* sp
7	-	Rods in pairs	+	nt	+	-	-	+	-	-	+	-	+	-	-	-	-	+	+	-	±	+	-	-	-	*Escherichia coli*
2	-	Rods in singles	+	nt	-	-	nt	-	+	-	-	+	+	+	+	+	+	+	+	+	+	+	+	-	+	*Klebsiella* sp

Key: nt: not tested, ±: variable, +: positive, -: negative

**Figure 1:**
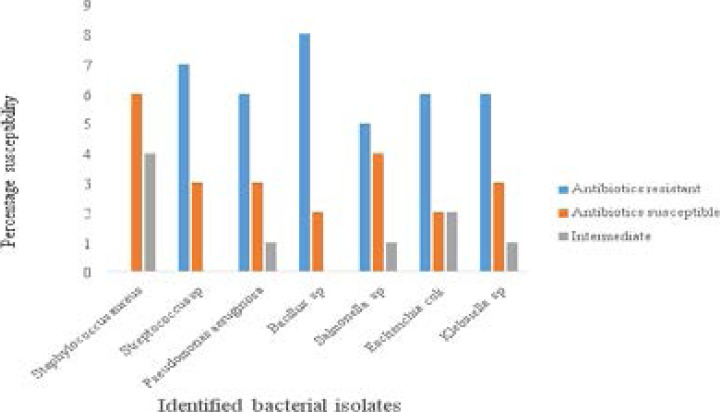
Antibiotic resistance pattern of the identified bacterial isolates

Figure 1 shows the percentage susceptibility pattern of the bacterial isolates to all the antibiotics used. The results revealed that all the identified bacterial isolates tested were resistant to more than 50% of the antibiotics used with the highest resistance rate observed in Augmentin, Ranicef, Levofloxacin and Ceforuxime while Nitrofurantoin, Ofloxacin, Ciprofloxacin and Imipenem recorded the least resistance rate. The proximate composition of soymilk showed that the samples had high moisture content and very low fibre and ash content as presented in Table 4.

**Table 4:** Proximate composition of soymilk samples (%) locally produced and sold in selected locations within Calabar Metropolis

Sample location	Moisture	Ash	Fibre	Protein	Fat	Carbohydrates
Watt Market	62.45	2.34	1.64	10.65	7.26	15.92
Akim Market	58.11	1.98	1.88	11.28	5.26	20.68
Marian Market	60.33	2.66	2.03	14.21	4.19	16.58
Atimbo Market	56.27	3.15	1.38	12.53	6.84	19.57
Goldie Market	61.11	2.83	2.18	16.27	5.18	12.43

## Discussion

Soymilk and soybean products have served as an important source of protein in the diet of millions of people for nearly 5,000 years. Its high nutrient value has made it so irresistible that it is recommended very highly by nutritionists as a substitute to cow milk. The increase in the consumption rate of soybean milk due to its high protein content has encouraged low scale production of the soymilk under house hold condition with little or no regard to the quality control measures^12^. The results of microbial load of soymilk samples locally produced in Calabar Metropolis revealed that the microbial count and the most probable number of coliform obtained exceeded the acceptable limit for both milk products and non-alcoholic beverages (<10^4^ cfu/mL This result agrees with the findings of other researchers^13^,^14^. The contamination of the soymilk is mostly as a result of poor handling, use of contaminated raw materials, unhygienic processing environment and lack of good manufacturing practices^12^.

The presence of *Pseudomonas aeruginosa* and other bacteria may be as a result of the composition of the soymilk and the handling processes during processing and storage^15^. Potentially harmful bacteria such as *Staphylococcus aureus, Pseudomonas aeruginosa* and *Streptococcus* sp. present in these products may cause diseases in the individuals that consume them. They may cause foodborne illnesses and quick spoilage of the food products leading to not only a public health risk but also an economic loss to producers and retailers.

Adebayo-Tayo *et al*^3^ and Nazim *et al*^16^ reported significantly higher fungal counts in locally produced soymilk products compared to fungal counts obtained in this study. The relatively higher fungal counts observed may indicate a contamination of the products during processing. This could cause the growth of potentially harmful fungi in these products, and may also lead to certain ill health when consumed17. The occurrence of *Aspergillus* sp. is a cause for concern as it can produce potentially harmful aflatoxins in the products when they are consumed^3^.

The antibiotic sensitivity results of the isolated bacteria from locally produced soymilk samples sold in Calabar Metropolis revealed that all the identified bacteria isolated were resistant to over 50% of the antibiotics used. This can correctly be described as superbugs – multidrug resistant bacteria. The occurrence of these superbugs presents a fundamental and serious threat to public health particularly as it concerns the treatment of infectious disease. The result provides more scientific data that strengthen the reports that multidrug resistance bacteria are on the rise globally.

Antibiotic sensitivity patterns recorded in this study are in contrast to the findings of Adelekan *et al*^17^ which reported an 80% sensitivity of isolates from industrially processed soybean products to Augumentin and Ciprofloxacin. Also, Daniyan and Ajibo^18^ reported that *Staphylococcus aureus* was resistant to Pefloxacin but susceptible to streptomycin, ciprofloxacin, ceftriaxone and cefuroxime. In a similar study by Agwa *et al*^19^ *Bacillus cereus* was shown to be resistant to erythromycin and ampiclox. Srinu *et al*^20^ also reported that *Escherichia coli* was sensitive to Streptomycin, Ceforuxime and ciprofloxacin, however, the findings of this study shows that *Escherichia coli* isolated was resistant to Ciprofloxacin and Ceforuxime. The ash content in this study was higher than that reported by Onweluzo and Nwakalor^21^ of 0.80% - 1.00%. However, it is within the range of 1.60% - 3.10 reported by Asuquo and Antai^1^. This may be due to the differences in particle size of the flour before sieving. The smaller the particle size of the flour, the more endosperms that will be exposed and as such more minerals will be extracted from soymilk. Also, protein content range of 10.650% - 16.27% in this study was quite higher than the values 6.78% - 7.76% reported by Liamngee *et al*^14^. This may be attributed to the varying degree of heat treatments which may have resulted in the destruction or inactivation of some amino acids for example cysteine.

## Conclusion

The results of this study revealed that soymilk samples which is highly consumed in Calabar, Cross River State were highly contaminated with microorganisms whose source may be traced to poor hygiene of producers, unsanitary conditions of processing equipment and raw materials. These finding shows varying public health concerns in the soymilk samples analysed. Antibiotic sensitivity pattern of the bacterial isolates indicates an occurrence of antibiotic resistant bacteria most likely due to the exposure of these products to several preservatives and sometimes unhygienic processes of production. The study shows an unacceptable microbiological quality of these products and a dire need for the strict implementation of HACCP protocols during production of these products to prevent the occurrence of food contamination, spread of antibiotic resistant organisms and other serious public health concerns.
